# Target prioritization and strategy selection for active case-finding of pulmonary tuberculosis: a tool to support country-level project planning

**DOI:** 10.1186/1471-2458-13-97

**Published:** 2013-02-02

**Authors:** Nobuyuki Nishikiori, Catharina Van Weezenbeek

**Affiliations:** 1 World Health Organization, Regional Office for the Western Pacific, Manila, Philippines; 2 Stop TB and Leprosy Elimination, Division of Combating Communicable Diseases, Regional Office for the Western Pacific, World Health Organization, United Nations Avenue, Manila, Philippines

## Abstract

**Background:**

Despite the progress made in the past decade, tuberculosis (TB) control still faces significant challenges. In many countries with declining TB incidence, the disease tends to concentrate in vulnerable populations that often have limited access to health care. In light of the limitations of the current case-finding approach and the global urgency to improve case detection, active case-finding (ACF) has been suggested as an important complementary strategy to accelerate tuberculosis control especially among high-risk populations. The present exercise aims to develop a model that can be used for county-level project planning.

**Methods:**

A simple deterministic model was developed to calculate the number of estimated TB cases diagnosed and the associated costs of diagnosis. The model was designed to compare cost-effectiveness parameters, such as the cost per case detected, for different diagnostic algorithms when they are applied to different risk populations. The model was transformed into a web-based tool that can support national TB programmes and civil society partners in designing ACF activities.

**Results:**

According to the model output, tuberculosis active case-finding can be a costly endeavor, depending on the target population and the diagnostic strategy. The analysis suggests the following: (1) Active case-finding activities are cost-effective only if the tuberculosis prevalence among the target population is high. (2) Extensive diagnostic methods (e.g. X-ray screening for the entire group, use of sputum culture or molecular diagnostics) can be applied only to very high-risk groups such as TB contacts, prisoners or people living with human immunodeficiency virus (HIV) infection. (3) Basic diagnostic approaches such as TB symptom screening are always applicable although the diagnostic yield is very limited. The cost-effectiveness parameter was sensitive to local diagnostic costs and the tuberculosis prevalence of target populations.

**Conclusions:**

The prioritization of appropriate target populations and careful selection of cost-effective diagnostic strategies are critical prerequisites for rational active case-finding activities. A decision to conduct such activities should be based on the setting-specific cost-effectiveness analysis and programmatic assessment. A web-based tool was developed and is available to support national tuberculosis programmes and partners in the formulation of cost-effective active case-finding activities at the national and subnational levels.

## Background

Global expansion of the WHO-recommended Stop TB strategy marked significant achievements in tuberculosis (TB) control, with 46 million patients successfully treated and seven million lives saved between 1995 and 2010 [1]. Despite the progress made, TB control today faces significant challenges. In many countries with declining incidence, TB tends to concentrate in vulnerable and marginalized populations that often have limited access to health care. There are a number of existing and emerging factors that contribute to the TB epidemic such as the human immunodeficiency virus (HIV), the widespread use of tobacco, the epidemics of noncommunicable diseases including diabetes mellitus, increasing flows of migration and widening socioeconomic disparities [2,3].

In addition, recent prevalence surveys have shown serious limitations of the current diagnostic approach. According to survey findings, approximately 40%–60% of TB patients would be ruled out through initial symptom screening under the routine programme setting (More precisely, 45% of TB patients in Viet Nam [4], 46% in South Africa [5] and 61% in Cambodia [6] would have been ruled out because they did not have conventional TB symptoms such as cough for more than two weeks). Similarly, smear microscopy can detect only a proportion (30% to 69%) of all confirmed cases [4-7]. Yet, most developing countries still have to rely on sputum smear microscopy for symptomatic patients who present to health facilities.

Active case-finding (ACF) is a special effort of the health care system to detect TB patients among people who do not seek care for TB symptoms [8,9]. In light of the limitations of the current case-finding approach and the global urgency to improve case detection, ACF has been suggested as an important complementary strategy to accelerate TB control [2,8-10].

To reflect the global attention, some international initiatives have been promoting various intensified case-finding activities. For example, the TB REACH grant mechanism managed by the Stop TB Partnership has been massively promoting country-level implementation of innovative case-finding strategies, including ACF. Nevertheless, comprehensive guidance is still lacking on ACF especially on which TB high-risk populations can be targeted and what diagnostic algorithms should be employed.

Adding to the difficulty, new diagnostic tools have been introduced in recent years [11]. Xpert MTB/RIF (Cepheid, USA), a fully automated real-time polymerase chain reaction assay, is one such tool. While these tools propose significant opportunities for improving TB control, countries are facing considerable challenges in introducing new tools into their national diagnostic networks and policies. Hence, there is an urgent need for general guidance on TB ACF, particularly on prioritizing target groups and selecting diagnostic strategies [2]. Since the feasibility and appropriateness of ACF depend largely on local settings, a careful cost assessment is crucial for rational policy development.

This paper aims at modelling different ACF diagnostic strategies while focusing on the estimated diagnostic yield of pulmonary TB cases and associated costs. A model was designed to estimate the cost-effectiveness parameters of ACF for different target populations under various epidemiological settings to support country-level planning. The model was transformed into a web-based tool which can support national TB programmes (NTPs) and civil society partners in formulating cost-effective and setting-specific ACF activities.

## Methods

### The model framework

**Figure 1:**
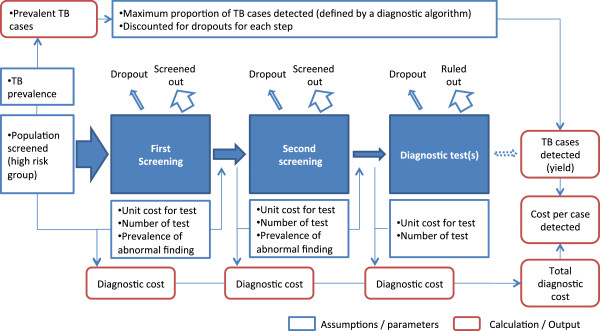
**Model framework.** The middle part represents the flow of a population screened until diagnosis. The upper part represents the calculation of a yield, i.e. total TB cases detected. The lower part explains the calculation of diagnostic cost for each step.

A simple deterministic model was designed to calculate the estimated pulmonary TB cases diagnosed and the associated direct costs for different diagnostic algorithms (Figure 1), with the parameters and assumptions listed in Table 1. The screening of TB involves multiple steps of several testing methods, usually consisting of one or two steps of screening tests followed by a confirmatory diagnosis with bacteriological tests.

**Table 1:** Key parameters and assumptions

**Parameter**	**Definition**	**Base value [range]**	**Remarks**
*TB prevalence among the general population and high-risk groups*			
*Prev*_*0*_	TB prevalence among the general population (per 100,000)	country specific	Estimated prevalence of all TB among general population based on the estimates in Global TB Control 2010, WHO.
PRi and Previ for Group i (i=1,2,…) where *Prev*_*i*_*= PR*_*i*_*× Prev*_*0*_	Assumed prevalence of pulmonary TB for each of the high-risk groups. Defined either by prevalence ratio to the general population or by direct estimate	PR_1_ = 1.0	Although not strictly accurate, risk-group prevalence parameters were set to allow general inference regarding some high-risk groups. (Group 1: General population; Group 2: Poor, urban dwellers; Group 3: Malnourished; Group 4: Diabetics; Group 5: TB contacts, and Group 6: Prisoners and/or other high-risk groups.) Note that these groups are only indicative. Users of the tool should define the prevalence parameters based on the best estimate for the local context.
		PR_2_ = 1.5	
		PR_3_ = 2.0	
		PR_4_ = 3.0	
		Prev_5_ = 4000/105	
		Prev_6_ = 6000/105	
*Proportion of abnormalities among screened subjects*			
Pr (symp) = *a* × *Prev*_*i*_*+ b (i=1,2,…)*	Proportion of TB symptomatics as a function of Prev_i_	a = 2.9829	Pr (symp) and Pr (X-ray) were expressed as a linear function of TB prevalence in the target population. The method was employed due to the observed linear trends in prevalence survey findings. Simple linear regression models were fit for multiple data points from prevalence surveys and resulted intercept and coefficient were used. * The overlap was assumed to be 20%, based on prevalence survey findings (References 5, 8). This parameter is required to calculate the combined suspects, i.e. {Pr (symp) ∪ Pr (X-ray)}.
		b = 0.0355	
Pr (X-ray) = *a* × *Prev*_*i*_*+ b (i=1,2,…)*	Proportion of subjects with X-ray abnormality as a function of Prev_i_	a = 3.0415	
		b = 0.0377	
Overlap*	{Pr (symp) ∩ Pr (X-ray)} divided by {Pr (symp) + Pr (X-ray)}	0.20	
*Proportional yields (Refer to Figure *2)			
	Proportions of prevalent TB cases who are		Sensitivity analysis was conducted for the ranges of PYsymp [0.3-0.5] and PYsmear [0.5-0.7], both are assumed to follow a uniform distribution.
*PYsymp*	TB symptomatic	0.40 [0.3-0.5]	
*PYsmear*	smear-positive	0.60 [0.5-0.7]	
*PYss*	TB symptomatic and smear-positive	0.24	Assuming PYsymp and PYsmear are independent, *PYss = PYsymp* × *PYsmear*
*PYsc*	TB symptomatic and smear-negative	0.16	*PYsc = PYsymp* × *(1-PYsmear)*
*PYxs*	Not TB symptomatic and smear-positive	0.36	*PYxs = (1-PYsymp)* × *PYsmear*
*PYxc*	Not TB symptomatic and smear-negative	0.24	*PYxc = (1-PYsymp)* × *(1-PYsmear)*
*Dropout rate for screening/diagnostic test*			
Symptom screening		0.00	A proportion of individuals who entered in a screening step but did not complete the step to receiving the result. The model assumed a high return rate for culture due to a long turnaround time for solid culture.
Sputum smear microscopy		0.02	
Chest X-ray (screening)		0.02	
Chest X-ray (diagnosis)		0.20	Similarly, the rate is high if the chest X-ray is used for diagnosis of smear-negative TB as it often requires some lead time for group diagnosis (e.g. TB diagnostic committees) as per national guidelines.
Sputum culture (solid)		0.15	
Xpert MTB/RIF		0.00	
Unit cost for screening/diagnostic test			
Symptom screening	in USD per person	0.02	The unit cost of screening and diagnostic tests in USD. They were meant to be direct unit cost excluding capital, equipment and human resources cost. These costs can be included in the analysis in the web-based tool if they are deduced to the cost per test.
Sputum smear microscopy	in USD per slide (three slides per person)	0.70	
Chest X-ray	in USD per test	3.00 [2.00-6.00]	
Sputum culture (solid)	in USD per test	5.00	
Xpert MTB/RIF	in USD per specimen	16.86	

The primary output of the model was the diagnostic cost per TB case detected, defined as the total diagnostic cost divided by the total number of TB cases diagnosed. The total diagnostic cost was derived from a simple step-by-step calculation of costs. The total number of TB cases diagnosed was calculated from the number of subjects screened, the TB prevalence among them, the proportion of prevalent TB cases diagnosed with a given set of diagnostic procedures (proportional yield) and the dropout rate. In the model, dropouts were defined as individuals who entered into a screening/diagnostic step but did not complete the whole step through to receiving the test result. In designing the model, TB prevalence was defined as the prevalence of bacteriologically-confirmed pulmonary TB.

### Diagnostic algorithms

Six diagnostic algorithms were predefined to examine their cost-effectiveness when applied to different target populations (Table 2). As a rapid guide, the strategies were constructed from Strategy 1 to Strategy 5 in an incremental manner from a simple, conservative approach to extensive approaches. Strategy 1, symptom screening followed by sputum microscopy, is similar to routine programme settings. Strategy 2 adds X-ray for diagnosis of smear-negative cases. Strategy 3 employs radiography screening for the entire target population, and Strategy 4 is a standard approach recommended by WHO for prevalence surveys [12]. Strategy 5 uses Xpert as the diagnostic test with the screening procedure of Strategy 4. Strategy 6 is the only strategy with two-step screening. The symptomatic subjects identified by the first screening undergo X-ray screening to reduce the number of subjects for the diagnostic test (Xpert). This algorithm reduces the number of Xpert tests at the cost of losing non-symptomatic TB patients at the first screening step.

**Table 2:** Diagnostic algorithms predefined for the model

**Strategy**	**Description**	**First screening**	**Second screening**	**Diagnostic test**	Maximum proportional yield*	**Example in Figure**2(d) condition***
**1**	Basic routine programme model	Symptom	–	Microscopy	PYss	24%
**2**	Strategy 1 + smear-negative diagnosis with X-ray	Symptom	–	Microscopy X-ray	PYss + part** PYsc	24–40%
**3**	X-ray + symptom screening	Symptom X-ray	–	Microscopy	PYss+PYxs+part** (PYsc+PYxc)	60%–100%
**4**	Prevalence survey model	Symptom X-ray	–	Microscopy Culture	PYss+PYsc+PYxs+PYxs	100%
**5**	Xpert in Strategy 4	Symptom X-ray	–	Xpert	PYss+PYsc+PYxs+PYxs	100%
**6**	Restrictive screening + Xpert	Symptom	X-ray	Xpert	PYss+PYsc	40%

* PY: Proportional yield (a proportion of prevalent TB cases by symptomatic and smear statuses). Please see Table 1.

** A diagnostic sensitivity of smear-negative TB can vary depending on the programmatic setup.

*** A maximum proportion of TB cases that can be diagnosed.

### Screening methods and prevalence of abnormality

All six diagnostic algorithms employ one of the following three screening approaches to identify subjects proceeding to the diagnostic tests: (i) TB symptomatic screening, i.e., typically, inquiring about the presence of prolonged cough for more than two weeks and/or haemoptysis; (ii) a combination of TB symptomatic screening and chest radiography, wherein the model assumes that individuals with TB symptoms and/or abnormal chest radiography would be ruled in; and (iii) a sequential application of symptom screening and chest radiography which identifies individuals with both TB symptoms and any abnormal chest radiography.

The first approach is bound to miss a substantial portion of TB patients (45%–61%) [4-6] who do not fulfil TB symptomatic criteria. The second approach is one of the most inclusive screening methods and has been recommended by WHO for TB prevalence surveys [12]. The basic idea of the third approach is to reduce the number of subjects who undergo an expensive diagnostic test. In our model, it was used only in Strategy 6, aiming to reduce the cost of tests using Xpert [11].

To calculate the number of subjects proceeding to the next step, the estimated prevalence of abnormality among the screened subjects was required. The information from prevalence surveys [4,6,12,13] was used to construct simple linear regression models in order to predict the prevalence of abnormalities as a function of TB prevalence (Table 1).

### Estimation of yields

**Figure 2:**
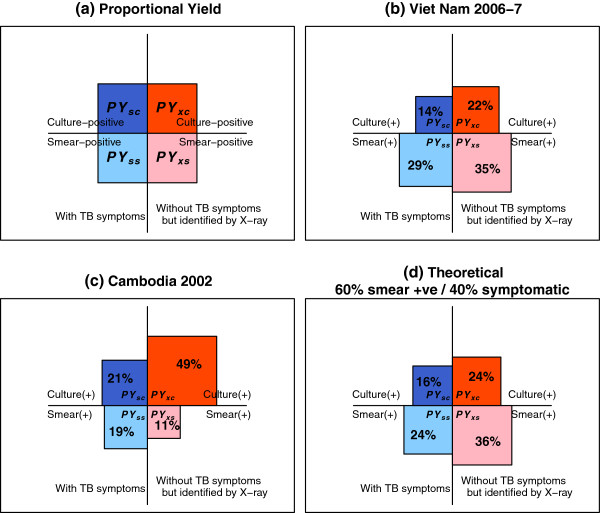
**Proportional yields.** (**a**) Proportions of prevalent TB cases are denoted as PYss, PYsc, PYxs and PYxc by TB symptoms and smear statuses where the total sum is 100% (Table 1). (**b**) Proportional yields based on the TB prevalence survey in Viet Nam.^7^ (**c**) Based on the TB prevalence survey in Cambodia.^4^ (**d**) Calculated by assuming that 40% of prevalent TB cases can be identified through symptomatic screening, 60% of TB cases are smear- positive and they are independently distributed. Note their similarity with (**b**).

A proportional yield, defined as the maximum proportion of prevalent TB cases diagnosed by a given diagnostic algorithm, was required to calculate the number of TB cases diagnosed. In other words, the proportional yield can be interpreted as the sensitivity of diagnostic methods in combination. The assumptions needed for estimating the proportional yield were distributions of prevalent TB cases by symptom and smear status as shown in Figure 2(a). These numbers can be derived from empirical data from prevalence surveys but, as shown in Figures 2(b) and 2(c), the survey findings were not fully consistent [4,6]. While more data are needed from other surveys, the yield can be theoretically calculated from two assumed parameters: the sensitivities of TB symptom screening and sputum smear microscopy. Figure 2(d) shows proportional yields that were calculated by assuming that 40% of TB cases were symptomatic and 60% were smear-positive. Based on a chosen set of the proportional yield, the number of TB cases diagnosed can be calculated by combining the segments in Table 2. Dropout rates defined in Table 1 were used to discount the eventual number of cases who could be diagnosed.

### TB prevalence among high-risk groups

The model intends to produce cost-effectiveness parameters for multiple risk groups at once so that the results can be compared to judge the relative importance of different risk groups. Estimated TB prevalence can be defined by two methods depending on the type of group. TB prevalence of some groups may be better defined as a prevalence ratio to the baseline prevalence (e.g. smokers) while other groups may have direct estimates of TB prevalence if relevant data is available (e.g. TB contacts, prisoners). For the model analysis, we arbitrarily defined the four TB risk groups by a prevalence ratio (PR=1.0, 1.5, 2.0 and 3.0) and two high-risk groups by prevalence estimates of 4000 and 6000 cases per 100,000 population (Table 1). A detailed discussion on the estimates of TB risk is not within the scope of this study. Nevertheless, literature is available on the estimates of differential risk of TB among various groups to make inference from the model outputs to a real-life scenario [14-18]. For the online tool, users are expected to set estimated prevalence parameters according to the local context and the model provides context-specific outputs to facilitate local level project planning.

### Output parameters

Under a given baseline of TB prevalence among the general population, the model estimates a diagnostic cost per case detected for each of the defined high-risk populations. Cost per case detected has been used in the global TB control community through two international initiatives: Fund for Innovative DOTS Expansion Through Local Initiatives to Stop TB (FIDELIS) by the International Union Against Tuberculosis and Lung Disease launched in 2004 [19], and TB REACH by the Stop TB Partnership launched in 2010 (http://www.stoptb.org/global/awards/tbreach). TB REACH generally recommends formulating a project within the overall budget of USD 350 per case detected and successfully treated. Since the model only calculates the direct diagnostic cost, not the costs associated with operation, logistics and treatment, USD 200 per case detected was arbitrarily used as a benchmark. Additional outputs include the Number Needed to Screen (NNS) and incremental yields in relation to the total costs incurred. Sensitivity analysis was conducted for the selected parameters for which values were less certain.

### Developing a tool to assist decision-making

The model was initially developed in Microsoft Excel, and summary outputs were utilized for formulating ACF projects in selected countries in the Western Pacific Region. After some upgrading, the tool was coded in the statistical package R (CRAN: the Comprehensive R Archive Network at http://cran.r-project.org) to conduct statistical analysis and simulations.

As an attempt to provide the model on a user-friendly and interactive platform, the tool was rewritten in JavaScript to be available on the Internet at http://www.innovationsinpublichealth.org.

## Results

### Incremental yields of TB cases detected

**Figure 3:**
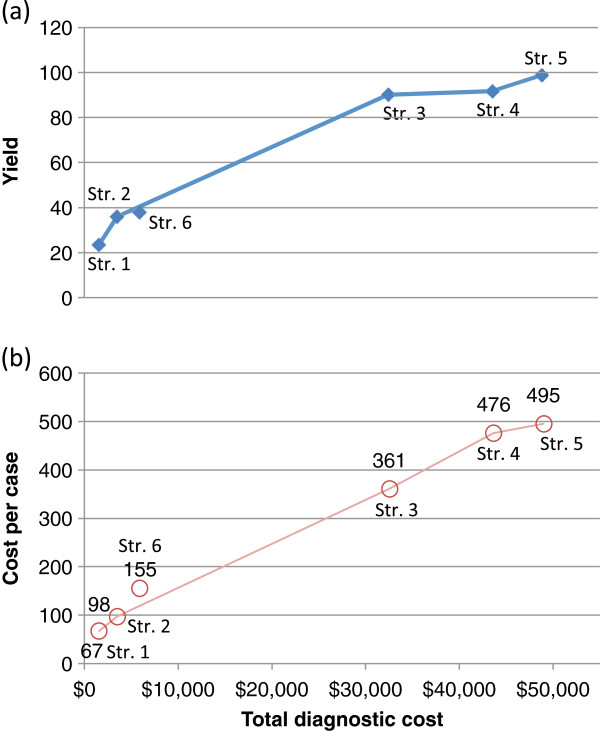
**Cost per case detected and incremental yield, by diagnostic strategy, in a hypothetical TB risk group of 10,000 population with TB prevalence of 1.0%.** The number of TB cases detected (**a**) and the cost per case detected are plotted against the total diagnostic cost. Each point represents a diagnostic strategy from strategies 1 to 6 defined in Table 2.

Figure 3(a) shows the yield of TB cases for each of the six diagnostic strategies against overall diagnostic cost under a hypothetical setting of 10,000 population with 1.0% of TB prevalence (1000 per 100,000 population). In general, the number of TB cases diagnosed increased as the diagnostic strategy moved from conservative to extensive (strategies 1 to 5) with an additional diagnostic cost at each step. Cost per case detected also linearly increased, except for Strategy 6, as shown in Figure 3(b).

Under the hypothetical setting, Strategy 1 diagnosed only 23.5 patients out of 100 prevalent TB cases in the population with a diagnostic cost of USD 1571. In terms of cost-effectiveness, this translates to USD 67 per case detected. With Strategy 2, a total of 36.1 patients (an additional 12.6 patients) were diagnosed with an overall diagnostic cost of USD 3530. Strategies 3 and 4 substantially increased the number of TB cases (90.2 and 91.7 patients, respectively), but the cost was substantially higher (more than tenfold, USD 32 535 and USD 43 655, respectively). Cost per case detected became USD 361 and USD 476 which is several times higher (i.e. less cost-effective) than the first two strategies. The increased cost for Strategy 3 was primarily due to X-ray examinations for the entire targeted population, and the difference between the two strategies (3 and 4) was the use of culture as a diagnostic test. The model assumed that Xpert yielded the same level as solid culture, but a relatively high dropout rate (15%) was set for culture considering the long turnaround time. As expected, Strategy 6 yielded a relatively small proportion of TB cases because the strategy applied the most restrictive approach in screening. However, it is important to note that this strategy seemed reasonably cost-effective with USD 155 per case detected, despite the higher cost of using of Xpert.

### Cost-effectiveness of ACF in relation to TB risk and diagnostic strategy

**Figure 4:**
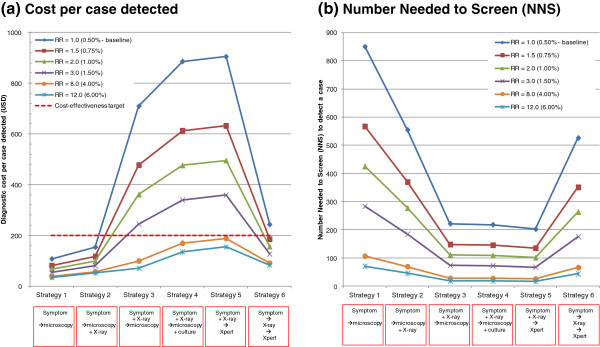
**Cost-effectiveness of tuberculosis active case-finding.** (**a**) Cost per case detected by diagnostic strategies and (**b**) the Number Needed to Screen (NNS) to detect a case in a hypothetical country with TB prevalence of 0.5% (500 per 100,000 population).

Figure 4(a) summarizes the cost-effectiveness of diagnostic strategies for different risk groups. Each of the six plotted lines represents a different risk group with PR of 1.0 (general population), 1.5 (e.g. poor urban communities), 2.0 (e.g. malnourished), 3.0 (e.g. diabetics), 8.0 (e.g. TB contacts) and 12.0 (e.g. prisoners or people living with HIV). The baseline TB prevalence was set at 0.5%, which is approximately the prevalence level for Bangladesh, the Democratic Republic of the Congo, Mozambique, Myanmar and the Philippines, and is lower than the prevalence for Cambodia and South Africa [1].

As observed in Figure 3, the general trend of decreasing cost-effectiveness with upgraded diagnostic strategies persisted for all risk populations. However, the cost per case detected was substantially reduced in a population with a very high prevalence such as TB contacts, prisoners or people living with HIV. In these very high-risk populations, the most extensive diagnostic strategies (such as the strategies 4 and 5) were still cost-effective (i.e. all strategies fell within a diagnostic cost per case detected of less than USD 200).

Another important finding was that conservative approaches which were similar to routine programme setups (strategies 1 and 2) were the most cost-effective strategies even though the yield was low. These strategies were cost-effective for almost all high-risk populations in TB high-burden countries.

The cost per case detected increased abruptly between Strategy 2 and Strategy 3 due to the application of X-rays for all subjects. With the given set of assumptions and using USD 200–250 as a cost-effectiveness target, TB prevalence around 1.5% was identified as the decision-making-point whether to employ X-ray screening for all. However, it requires context-specific analysis because the cost-effectiveness of Strategy 3 appears sensitive to the unit cost of X-ray which varies among different countries and localities. This issue was further examined in sensitivity analyses.

### Number Needed to Screen (NNS)

The Number Needed to Screen, defined as the total number of subjects screened divided by the number of TB cases detected, is presented in Figure 4(b). Obviously, the higher the TB prevalence, the lower the NNS. However, even within the same risk group, the NNS significantly differed depending on the diagnostic strategy. Under the assumptions for Figure 4, NNS went up to several hundred for the low-yield strategies (1 or 2), but the diagnostic cost was actually very low. Conversely, the NNS was minimized by applying extensive diagnostic strategies that yielded more cases but were costly. Due to this inverse relationship between the NNS and the diagnostic cost, a low NNS would not necessarily guarantee the feasibility and cost-effectiveness of ACF. The reality seems the other way round - inexpensive strategies (1 and 2) were almost always cost-effective even though the NNS was very high.

### Sensitivity analysis

**Figure 5:**
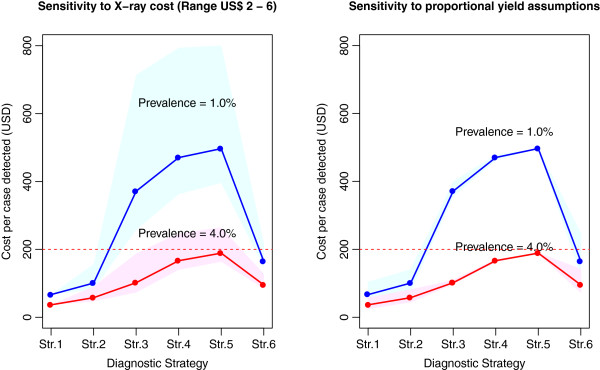
**Sensitivity analysis for X-ray cost and proportional yield assumptions.** Cost per case detected for target populations with TB prevalence of 1.0% and 4.0% under uniform distribution of the values for (**a**) X-ray cost, and (**b**) proportional yield assumptions. The parameter ranges are shown in Table 1.

The sensitivity of the cost per case detected was examined under varied parameter conditions. Figure 5(a) presents the result when the unit cost of X-ray uniformly varied between USD 2 and 6. As expected, the cost per case detected varied significantly for strategies 3, 4 and 5. Importantly, the output was more robust under the prevalence of 4.0% than under 1.0%.

Another less certain set of assumptions was the proportional yield. The uncertainty bound was obtained by uniformly, simultaneously and independently varying the proportion of symptomatic patients between 30% and 50% and the proportion of smear-positive patients between 50% and 70% (Table 1). As a result, Figure 5(b) shows that the model output was reasonably robust against the proportional yield assumptions, which minimized the issue of uncertainty due to insufficient empirical data.

## Discussion

Our analysis reconfirms that TB ACF can be a costly undertaking, depending on the target population and the diagnostic strategy used. Therefore, the prioritization of proper target populations and careful selection of cost-effective diagnostic strategies are critical prerequisites for launching any rational ACF activities.

There seem to be several important conditions under which ACF can be cost-effective and, in such circumstances, it can contribute to a significant increase in case detection. First, TB prevalence among a target population is a very important determinant of cost-effectiveness. The higher the TB prevalence among the target population, the more TB cases detected thus contributing to better cost-effectiveness.

Second, using the conventional DOTS approach, symptom screening followed by smear microscopy (Strategy 1) was found to be almost always cost-effective as the unit cost of tests is inexpensive. Although the yield is very low due to low sensitivity, the strategy is always an option for a population with different TB risks.

Third, in designing ACF activities, a critical decision to make is to determine whether all subjects should undergo X-ray screening. The cost per case detected for strategies 3, 4 and 5 was sensitive to the unit cost of X-ray which generally ranges between USD 2 and USD 6, in accordance with the country context. Therefore, it is important to carefully assess the cost-effectiveness of ACF by using accurate local cost information including human resources, as well as infrastructural and logistics expenses. This was part of the reason for providing an online tool for national stakeholders to examine the cost-effectiveness indicators according to their local settings. To be noted, digital X-ray technologies could substantially reduce the unit cost of X-ray (without printing the films), and substantially expand the potential of X-ray screening for ACF.

As with any model analysis, the current approach carries several limitations. The model assumes a number of parameters based on available information including the prevalence of test abnormalities, proportional yields and unit costs. Although we used the best available information for these assumptions, our source of information might be biased towards Asian settings due to the availability of such data. Including data from other parts of the world might be needed as a future development of the model. To address the uncertainly around the assumptions, we conducted sensitivity analysis of the model output. It is important to note that the model output was quite robust against the diagnostic yield assumptions, which support the general observation described above.

The cost information was limited to direct diagnostic costs that represent a part of the minimum necessary costs for ACF activities. In reality, other operational costs such as those involving human resources and logistics should also be considered in any project planning. In view of this, the model output should be used to check whether the proposed diagnostic strategy is worth further consideration or if its diagnostic cost alone is too prohibitive to consider further project planning.

Besides cost per case detected employed in our analysis, there would be other important cost-effectiveness and cost-utility indicators such as deaths averted, secondary cases prevented and disability-adjusted life years saved. All of these are important indicators in the context of TB ACF. For example, preventing secondary infection through early case-finding [20,21] might be one of the major benefits of ACF in reducing the TB burden in a community. However, assessing these benefit indicators requires many additional assumptions for which information is scarce. Moreover, the evidence base is still insubstantial to support any epidemiological impact of ACF though, theoretically, it is expected to exist.

For the reasons stated above, it is justifiable to limit the scope to the simple but robust cost-effectiveness calculation. Our intention was to support national TB programmes to formulate various ACF initiatives rather than to model the epidemiological impact of ACF. Nevertheless, we believe the model and our online tool can contribute to the debate on conditions required for cost-effective ACF and the selection of diagnostic strategies for different target groups.

TB ACF is not a new intervention. It has been extensively used in many parts of the world, sometimes involving mass radiological screening [8]. As our model shows, these ACF activities can be cost-effective only against the backdrop of high TB prevalence in the society or when targeting a TB high-risk population. This explains the fact that many industrialized countries discontinued mass population screening when the TB prevalence among the general population decreased. However, in countries with a high burden of TB and a well-established basic DOTS programme, there is a renewed interest in ACF as a complementary strategy to increase case detection [2].

Many studies documented several important benefits of ACF over routine passive case-finding. ACF can detect a substantial portion of undiagnosed TB patients much earlier than passive case-finding while their bacterial load is low [20-23]. Consequently, it also contributes to reducing transmission by shortening the duration of infectiousness [24,25]. Moreover, ACF would potentially play an important role to address health inequities. ACF can specifically target and benefit vulnerable segments of the population such as the elderly, the poor, and the marginalized [15,20,22,23].

Our online tool provides some essential information on TB ACF that would help national TB programme managers and partners make decisions on priority target populations and cost-effective diagnostic strategies. In the current culture of information technology, the concept of an interactive, online tool for context-specific decision-making is not novel. However, it is also true that public health programmes are not benefiting as much from the full potential of available technologies as the private sector. We aimed at providing a model of an interactive tool that contributes to the national and subnational levels of planning for public health activities. Real-time user experience and feedback will help us further improve the model which may, overtime, initiate an innovative way to build up and refine public health policy guidance.

## Conclusions

The prioritization of appropriate target populations and careful selection of cost-effective diagnostic strategies are critical prerequisites for rational active case-finding activities. A decision to conduct such activities should be based on the setting-specific cost-effectiveness analysis and programmatic assessment. A web-based tool was developed and is available to support national tuberculosis programmes and partners in the formulation of cost-effective active case-finding activities at the national and subnational levels.

## Competing interests

The authors declare that they have no competing interests.

## Authors’ contributions

NN developed the initial concept, constructed the model and conducted analysis. CVW provided overall guidance for the model. NN and CVW jointly interpreted the results and drafted the manuscript. The authors alone are responsible for the views expressed in this publication and they do not necessarily represent the decisions or policies of the World Health Organization. Both authors read and approved the final manuscript.

## Pre-publication history

The pre-publication history for this paper can be accessed here:

http://www.biomedcentral.com/1471-2458/13/97/prepub

## References

[B1] World Health Organization Global tuberculosis control: WHO report 2011 2011 Geneva: World Health Organization 978 92 4 156438 0.

[B2] Lönnroth K Castro KG Chakaya JM Chauhan LS Floyd K Glaziou P Raviglione MC Tuberculosis control and elimination 2010–50: cure, care, and social development Lancet 2010 375 1814 1829 10.1016/S0140-6736(10)60483-7 20488524

[B3] Lönnroth K Jaramillo E Williams BG Dye C Raviglione M Drivers of tuberculosis epidemics: the role of risk factors and social determinants Soc Sci Med 2009 68 12 2240 2246 10.1016/j.socscimed.2009.03.041 19394122

[B4] Hoa NB Sy DN Nhung NV Tiemersma EW Borgdorff MW Cobelens FG National survey of tuberculosis prevalence in Viet Nam Bull World Health Organ 2010 88 273 280 10.2471/BLT.09.067801 20431791 PMC2855599

[B5] den Boon S White NW van Lill SW Borgdorff MW Verver S Lombard CJ Bateman ED Irusen E Enarson DA Beyers N An evaluation of symptom and chest radiographic screening in tuberculosis prevalence surveys Int J Tuberc Lung Dis 2006 10 8 876 882 16898372

[B6] Ministry of Health Cambodia National Tuberculosis Prevalence Survey Cambodia, 2002 2005 Phnom Penh: Ministry of Health Cambodia

[B7] Corbett EL Bandason T Duong T Dauya E Makamure B Churchyard GJ Williams BG Munyati SS Butterworth AE Mason PR Comparison of two active case-finding strategies for community-based diagnosis of symptomatic smear-positive tuberculosis and control of infectious tuberculosis in Harare, Zimbabwe (DETECTB): a cluster-randomised trial Lancet 2010 376 9748 1244 1253 10.1016/S0140-6736(10)61425-0 20923715 PMC2956882

[B8] Golub JE Mohan CI Comstock GW Chaisson RE Active case finding of tuberculosis: historical perspective and future prospects Int J Tuberc Lung Dis 2005 9 11 1183 1203 16333924 PMC4472641

[B9] Borgdorff MW Floyd K Broekmans JF Interventions to reduce tuberculosis mortality and transmission in low- and middle-income countries Bull World Health Organ 2002 80 217 227 11984608 PMC2567749

[B10] Murray CJ Salomon JA Expanding the WHO tuberculosis control strategy: rethinking the role of active case-finding Int J Tuberc Lung Dis 1998 2 9 Suppl 1 S9 S15 9755959

[B11] World Health Organization Rapid implementation of the Xpert MTB/RIF diagnostic test: technical and operational ‘How-to’; practical considerations 2011 Geneva: World Health Organization 978 92 4 150156 9.

[B12] World Health Organization Tuberculosis prevalence surveys: a handbook 2011 Geneva: World Health Organization 978 92 4 154816 8.

[B13] Department of Health and Tropical Disease Foundation 2007 Nationwide Tuberculosis Prevalence Survey 2008 Manila: Department of Health Philippines and Tropical Disease Foundation

[B14] Jeon CY Murray MB Diabetes mellitus increases the risk of active tuberculosis: a systematic review of 13 observational studies PLoS Med 2008 5 7 e152 10.1371/journal.pmed.0050152 18630984 PMC2459204

[B15] Lonnroth K Jaramillo E Williams B Dye C Raviglione M Blas E, Kurup AS Tuberculosis: the role of risk factors and social determinants Equity, Social Determinants and Public Health Programmes 2010 Geneva: World Health Organization 219 241 978 92 4 156397 0.

[B16] Lonnroth K Williams BG Cegielski P Dye C A consistent log-linear relationship between tuberculosis incidence and body mass index Int J Epidemiol 2010 39 1 149 155 10.1093/ije/dyp308 19820104

[B17] Morrison J Pai M Hopewell PC Tuberculosis and latent tuberculosis infection in close contacts of people with pulmonary tuberculosis in low-income and middle-income countries: a systematic review and meta-analysis Lancet Infect Dis 2008 8 6 359 368 10.1016/S1473-3099(08)70071-9 18450516

[B18] Slama K Chiang CY Enarson DA Hassmiller K Fanning A Gupta P Ray C Tobacco and tuberculosis: a qualitative systematic review and meta-analysis Int J Tuberc Lung Dis 2007 11 10 1049 1061 17945060

[B19] Rusen ID Enarson DA FIDELIS-innovative approaches to increasing global case detection of tuberculosis Am J Public Health 2006 96 1 14 16 10.2105/AJPH.2004.056762 16317206 PMC1470445

[B20] Eang MT Satha P Yadav RP Morishita F Nishikiori N van-Maaren P Weezenbeek CL Early detection of tuberculosis through community-based active case finding in Cambodia BMC Public Health 2012 12 469 10.1186/1471-2458-12-469 22720878 PMC3489610

[B21] Lin X Chongsuvivatwong V Lin L Geater A Lijuan R Dose-response relationship between treatment delay of smear-positive tuberculosis patients and intra-household transmission: a cross-sectional study Trans R Soc Trop Med Hyg 2008 102 8 797 804 10.1016/j.trstmh.2008.04.027 18513768

[B22] den Boon S Verver S Lombard CJ Bateman ED Irusen EM Enarson DA Borgdorff MW Beyers N Comparison of symptoms and treatment outcomes between actively and passively detected tuberculosis cases: the additional value of active case finding Epidemiol Infect 2008 136 10 1342 1349 18177518 10.1017/S0950268807000106 PMC2870736

[B23] Yimer S Holm-Hansen C Yimaldu T Bjune G Evaluating an active case-finding strategy to identify smear-positive tuberculosis in rural Ethiopia Int J Tuberc Lung Dis 2009 13 11 1399 1404 19861013

[B24] Verver S Bwire R Borgdorff MW Screening for pulmonary tuberculosis among immigrants: estimated effect on severity of disease and duration of infectiousness Int J Tuberc Lung Dis 2001 5 5 419 425 11336272

[B25] Verver S van Soolingen D Borgdorff MW Effect of screening of immigrants on tuberculosis transmission Int J Tuberc Lung Dis 2002 6 2 121 129 11931410

